# Association Between the Number of Invasive Tumor Foci and Axillary Nodal Burden in Multifocal and Multicentric Breast Cancer

**DOI:** 10.3390/curroncol33100638

**Published:** 2026-10-21

**Authors:** Bulent Yildiz, Yasemin Sagdic Karateke, Duygu Bayir Garbioglu, Metin Demir

**Affiliations:** Department of Medical Oncology, Faculty of Medicine, Eskisehir Osmangazi University, 26040 Eskisehir, Türkiye; yasmins88@hotmail.com (Y.S.K.); duygubayir@hotmail.com (D.B.G.); dr_metindemir@hotmail.com (M.D.)

**Keywords:** breast cancer, multifocal breast cancer, multicentric breast cancer, number of tumor foci, axillary lymph node, nodal burden, disease-free survival

## Abstract

Multifocal and multicentric (MF/MC) breast cancers are associated with increased axillary lymph node involvement, but the relevance of the number of invasive tumor foci is less clear. This retrospective study included 1087 patients with non-metastatic invasive breast cancer who underwent definitive surgery between 2010 and 2022 without prior neoadjuvant therapy. Patients were grouped as having one, two, or three or more invasive foci; the three-or-more-foci category was used pragmatically to avoid small subgroups at higher focus counts rather than as a data-derived threshold. Multivariable logistic and Cox regression analyses were used to evaluate advanced nodal disease (N2/N3) and disease-free survival (DFS), respectively. N2/N3 disease was present in 18.4%, 19.0%, and 38.2% of patients, respectively (*p* < 0.001). Three or more foci were independently associated with N2/N3 disease (OR 2.81, 95% CI 1.65–4.79; *p* < 0.001), whereas two foci were not. At a median follow-up of 113.1 months, 10-year DFS rates were 77.0%, 82.5%, and 55.7%, respectively (log-rank *p* = 0.003). Three or more foci remained associated with poorer DFS after multivariable adjustment (HR 1.58, 95% CI 1.07–2.33; *p* = 0.022), whereas two foci were not. A statistically significant difference in OS was not demonstrated (log-rank *p* = 0.088), although the study may have had limited power for this endpoint. Greater nodal burden and poorer DFS were mainly observed in patients with three or more invasive foci. Focus number may help characterize this heterogeneity within MF/MC disease, although its value beyond total invasive tumor burden remains uncertain.

## Simple Summary

Multifocal and multicentric breast cancers are generally considered as a single group, although the number of invasive tumor foci may vary considerably among patients. In this study of 1087 patients with surgically treated invasive breast cancer, axillary nodal burden was similar in patients with one or two invasive foci but was substantially greater in those with three or more foci. Patients with three or more foci were more likely to have advanced nodal disease and also had poorer disease-free survival, whereas these associations were not observed in patients with two foci. The three-or-more-foci group was used as a pragmatic analytical category to avoid small subgroups at higher focus counts and should not be interpreted as a biologically established threshold. No statistically significant difference in overall survival was demonstrated, although the study may have had limited power for this endpoint. Further studies are needed to determine whether these associations reflect focus number itself or greater total invasive tumor burden.

## 1. Introduction

Multifocal (MF) and multicentric (MC) breast cancers represent distinct patterns of multiple ipsilateral breast tumors. Although definitions vary across studies, MF disease generally refers to separate tumor foci within the same quadrant, whereas MC disease refers to tumor foci located in different quadrants. Their reported frequency varies depending on the definitions used and the methods of imaging and pathological assessment [1,2].

Axillary lymph node involvement remains an important determinant of stage and prognosis in invasive breast cancer. Several studies have reported higher rates of axillary lymph node metastasis in patients with MF/MC tumors than in those with unifocal disease [3,4,5]. This association has also been observed within comparable tumor size categories, suggesting that tumor multiplicity may be associated with nodal involvement beyond the effect of dominant tumor size [3,4].

In MF/MC breast cancer, the T category is based on the largest invasive focus rather than the combined dimensions of all invasive foci [4,6,7]. However, the largest focus may not fully reflect the extent of multifocal disease, and alternative approaches using aggregate tumor measurements have been investigated [4,8].

Most previous studies have compared MF/MC tumors with unifocal breast cancers as categorical groups [3,4,5,9]. The number of invasive tumor foci itself has received less attention. It remains unclear whether an increasing number of invasive foci is associated with greater axillary nodal burden, independent of the size of the largest focus and other clinicopathological characteristics.

The present study aimed to evaluate the association between MF/MC disease, the number of invasive tumor foci, and axillary nodal burden in patients with surgically treated invasive breast cancer. We specifically examined the relationships between the number of invasive tumor foci, metastatic lymph node count, and advanced nodal disease (N2/N3), and whether these associations were independent of tumor size and other clinicopathological factors. We also evaluated the association between the number of invasive tumor foci and long-term survival outcomes.

## 2. Materials and Methods

### 2.1. Study Population and Data Collection

This retrospective study included patients with invasive breast cancer who underwent definitive breast surgery and were followed at the Department of Medical Oncology, Eskisehir Osmangazi University Faculty of Medicine, between January 2010 and December 2022. Clinical and pathological data were obtained from electronic medical records and pathology reports.

Patients aged ≥18 years with histologically confirmed invasive breast cancer, no distant metastasis at diagnosis, and available data on tumor focality, tumor size, and axillary lymph node status were eligible. Patients with carcinoma in situ without an invasive component, metastatic disease at diagnosis, previous neoadjuvant systemic therapy, or insufficient pathological data were excluded.

Recorded variables included age, menopausal status, tumor laterality and location, surgical procedure, histological type and grade, tumor size, lymphatic, vascular and perineural invasion, extracapsular extension, ductal carcinoma in situ (DCIS) and lobular carcinoma in situ (LCIS) components, ER, PR, HER2 status, and Ki-67. HER2 status was determined by immunohistochemistry (IHC), with fluorescence in situ hybridization (FISH) performed in cases with equivocal (IHC 2+) results. Data on adjuvant chemotherapy, radiotherapy, anti-HER2 therapy, endocrine therapy, distant metastasis, follow-up, and survival were also collected.

### 2.2. Definition of Tumor Focality

Tumor focality and the number of invasive tumor foci were retrospectively obtained primarily from the original pathology reports. Historical cases were not systematically re-reviewed for the purposes of this study. Available radiological findings were used as supplementary information when incorporated into the routine pathological assessment. After gross sectioning of the surgical specimen, tumor-containing slices were mapped and sampled to assess the spatial relationship between invasive tumor areas. In routine pathological assessment, separate invasive foci were considered distinct when they were separated by at least 5 mm and by intervening non-neoplastic breast parenchyma. Fibrotic separation alone, without intervening non-neoplastic breast tissue, was not considered sufficient to define a separate focus. Tumors were classified as unifocal, multifocal (MF), or multicentric (MC) according to the pathology records. MF disease was defined as the presence of two or more separate invasive tumor foci within the same quadrant, whereas MC disease was defined as two or more invasive tumor foci involving different quadrants of the same breast. For the primary comparisons, MF and MC tumors were combined into an MF/MC group and compared with unifocal tumors, as the main analysis focused on invasive focus number rather than anatomical distribution. Separate exploratory analyses were performed within the MF and MC groups.

In patients with MF/MC disease, the number of invasive tumor foci was recorded separately. Four patients had bilateral breast cancer. For these patients, the breast with more invasive foci was used for analysis. Focus number, tumor size, grade, receptor status, and axillary nodal data were taken from that breast; foci from the two breasts were not added together. Tumor size was defined according to the largest invasive tumor focus rather than the aggregate diameter of multiple foci.

### 2.3. Assessment of Axillary Nodal Burden

Axillary nodal status was assessed using the surgical pathology findings. The total number of examined lymph nodes and the number of metastatic lymph nodes were recorded for each patient. Pathological nodal stage was categorized as N0, N1, N2, or N3.

Axillary nodal burden was evaluated in several ways. The presence of any nodal metastasis and the number of metastatic lymph nodes were assessed, and pathological nodal stage was examined across focality groups. For the primary analysis, advanced nodal burden was defined as N2/N3 disease, with N0/N1 disease serving as the reference category. N2/N3 was chosen as the primary endpoint to distinguish advanced nodal burden from absent or limited nodal involvement. Any nodal involvement was evaluated separately.

### 2.4. Statistical Analysis

Comparisons between patients with unifocal and multifocal/multicentric (MF/MC) tumors were performed using the Mann–Whitney U test for continuous variables and the Pearson chi-square test or appropriate exact tests, including the Fisher–Freeman–Halton exact test, for categorical variables.

For analysis, patients were categorized according to the number of invasive tumor foci as one focus, two foci, or three or more foci. Patients with three or more foci were grouped together to avoid small subgroups at higher focus counts and obtain more stable estimates, while retaining a separate category for patients with two foci. Continuous variables across these groups were compared using the Kruskal–Wallis test, while categorical variables were compared using the chi-square test or exact tests, as appropriate. When the overall comparison was statistically significant, pairwise post-hoc comparisons were performed with Holm adjustment for multiple comparisons. Exploratory analyses compared patients with two versus three or more foci separately within the MF and MC groups. N2/N3 proportions were compared using Fisher’s exact test, and DFS was evaluated using univariable Cox regression.

Univariable logistic regression analyses were performed to identify factors associated with advanced axillary nodal burden. A multivariable logistic regression model was used to evaluate whether the number of invasive tumor foci was independently associated with advanced nodal disease after adjustment for age, tumor size, histological grade, histological type, lymphatic invasion, vascular invasion, estrogen receptor (ER) status, progesterone receptor (PR) status, and HER2 status. Results are reported as odds ratios (ORs) with 95% confidence intervals (CIs). As a sensitivity analysis, the same multivariable logistic regression model for N2/N3 disease was repeated in patients with at least 10 examined lymph nodes to assess the potential effect of axillary sampling on nodal classification.

Disease-free survival (DFS) was defined as the time from definitive breast surgery to the first occurrence of locoregional recurrence, distant metastasis, or death from any cause, whichever occurred first. Overall survival (OS) was defined as the time from definitive breast surgery to death from any cause. Patients without the respective event were censored at the date of last follow-up. Median follow-up was estimated using the reverse Kaplan–Meier method. Survival distributions according to the number of invasive tumor foci were estimated using the Kaplan–Meier method and compared using the log-rank test. Confidence intervals for the 5- and 10-year survival estimates were calculated using Greenwood’s variance with a log–log transformation. Univariable and multivariable Cox proportional hazards regression analyses were performed to evaluate factors associated with DFS. A univariable Cox regression analysis was also performed for OS according to the number of invasive tumor foci. The multivariable model included the number of invasive tumor foci, age, tumor size, histological grade, histological type, lymphatic invasion, vascular invasion, ER status, PR status, HER2 status, and advanced nodal disease (N2/N3).

In a sensitivity analysis, the DFS model was additionally adjusted for adjuvant chemotherapy, radiotherapy, endocrine therapy, trastuzumab use, and surgery type. Adjuvant treatments were coded as received or not received, and surgery as breast-conserving surgery or mastectomy. A second sensitivity analysis used a reduced Cox model including focus number, age, tumor size, histological grade, and N2/N3 disease. Results are reported as hazard ratios (HRs) with 95% CIs. The proportional hazards assumption was assessed using time-dependent covariates and log-minus-log survival plots, as appropriate. A time-dependent sensitivity analysis was performed for PR status because of a violation of this assumption, by adding a PR × ln(time) interaction term to the multivariable Cox model, with time measured in months. In exploratory analyses, the actual number of invasive foci was entered as a continuous variable in the multivariable logistic and Cox models, retaining the covariates used in the primary analyses. Nonlinearity was assessed using restricted cubic splines with knots at one, two, and three foci, comparing the spline and linear models by likelihood ratio tests. Statistical analyses were performed using IBM SPSS Statistics for Windows, version 27.0 (IBM Corp., Armonk, NY, USA). Restricted cubic spline analyses were performed using Python 3.12 (Python Software Foundation, Wilmington, DE, USA) and SciPy 1.17 (SciPy Community). The variables included in the regression models were complete, and all 1087 patients were analysed in the primary models. Ki-67 was missing in 299 patients (27.5%) and was not included in the models to avoid excluding these patients. No missing values were imputed. A two-sided *p* value < 0.05 was considered statistically significant.

### 2.5. Ethics

The study was conducted in accordance with the principles of the Declaration of Helsinki and was approved by the Eskisehir Osmangazi University Non-Interventional Clinical Research Ethics Committee (approval no: 86; date: 21 July 2026). The data cut-off date was 10 August 2026. The requirement for informed consent was waived by the Ethics Committee due to the retrospective nature of the study.

## 3. Results

A total of 1087 patients were included; 898 (82.6%) had unifocal tumors and 189 (17.4%) had multifocal/multicentric (MF/MC) disease. Baseline clinicopathological characteristics according to tumor focality are summarized in Table 1. Patients with MF/MC disease were younger than those with unifocal tumors, and significant differences were also observed in menopausal status, tumor laterality, surgical procedure, histological type, presence of a DCIS component, and HER2 status. No significant differences were observed in tumor size, histological grade, lymphatic or vascular invasion, perineural invasion, LCIS component, ER or PR status, or Ki-67.

Axillary lymph node involvement was more frequent in patients with MF/MC disease than in those with unifocal tumors (60.3% vs. 51.0%, *p* = 0.020). The median number of metastatic lymph nodes was 1 in both groups, although the distribution differed between the groups [1 (0–4) vs. 1 (0–2), *p* = 0.002]. The number of examined lymph nodes was similar [17 (12–24) vs. 17 (11–24), *p* = 0.696].

The distribution of pathological nodal stage differed between unifocal and MF/MC tumors (*p* = 0.016). N2/N3 disease was present in 28.0% of patients with MF/MC disease and 18.4% of those with unifocal tumors (*p* = 0.003). Extracapsular extension was also more common in the MF/MC group (33.9% vs. 24.9%, *p* = 0.012) (Table 2).

Of the 1087 patients, 898 had one invasive focus, 100 had two foci, and 89 had three or more foci. Among the 189 patients with MF/MC disease, 160 had MF and 29 had MC tumors. Two foci were present in 85 MF and 15 MC tumors, while three or more foci were present in 75 MF and 14 MC tumors. The median number of metastatic lymph nodes was 1 (0–2), 1 (0–3), and 2 (0–8), respectively (*p* < 0.001). Axillary lymph node involvement was observed in 51.0%, 54.0%, and 67.4% of patients, respectively (*p* = 0.012).

N2/N3 disease was present in 18.4% of patients with one focus, 19.0% of those with two foci, and 38.2% of those with three or more foci (*p* < 0.001). Extracapsular extension occurred in 24.9%, 24.0%, and 44.9% of patients, respectively (*p* < 0.001). Pairwise post-hoc analyses with Holm adjustment showed no significant differences between patients with one and two foci, whereas patients with three or more foci generally had greater nodal burden than the other groups (Table 3).

Within the MF group, N2/N3 disease was present in 18/85 patients (21.2%) with two foci and 29/75 (38.7%) with three or more foci (Fisher’s exact *p* = 0.023). The corresponding proportions in the MC group were 1/15 (6.7%) and 5/14 (35.7%), respectively (*p* = 0.080).

In univariable analysis, three or more invasive tumor foci were associated with advanced nodal disease (OR 2.75, 95% CI 1.73–4.35; *p* < 0.001), while two foci were not (OR 1.04, 95% CI 0.61–1.77; *p* = 0.878). Tumor size, histological grade, lymphatic invasion, vascular invasion, PR status, and HER2 status were also associated with N2/N3 disease (Table 4).

After adjustment for clinicopathological factors, three or more invasive foci remained independently associated with advanced nodal disease (OR 2.81, 95% CI 1.65–4.79; *p* < 0.001). Two foci were not independently associated with N2/N3 disease (OR 1.29, 95% CI 0.73–2.29; *p* = 0.383). Larger tumor size, grade 2 or 3 disease, and lymphatic invasion were also independently associated with N2/N3 disease (Table 4).

Overall, 218 patients (20.1%) had fewer than 10 examined lymph nodes: 184/898 (20.5%) with one focus, 21/100 (21.0%) with two foci, and 13/89 (14.6%) with three or more foci. In the sensitivity analysis restricted to 869 patients with at least 10 examined lymph nodes, three or more invasive foci remained associated with N2/N3 disease (adjusted OR 2.56, 95% CI 1.45–4.50; *p* = 0.001), whereas two foci were not significantly associated with N2/N3 disease (adjusted OR 1.31, 95% CI 0.72–2.41; *p* = 0.380).

**Figure 1:**
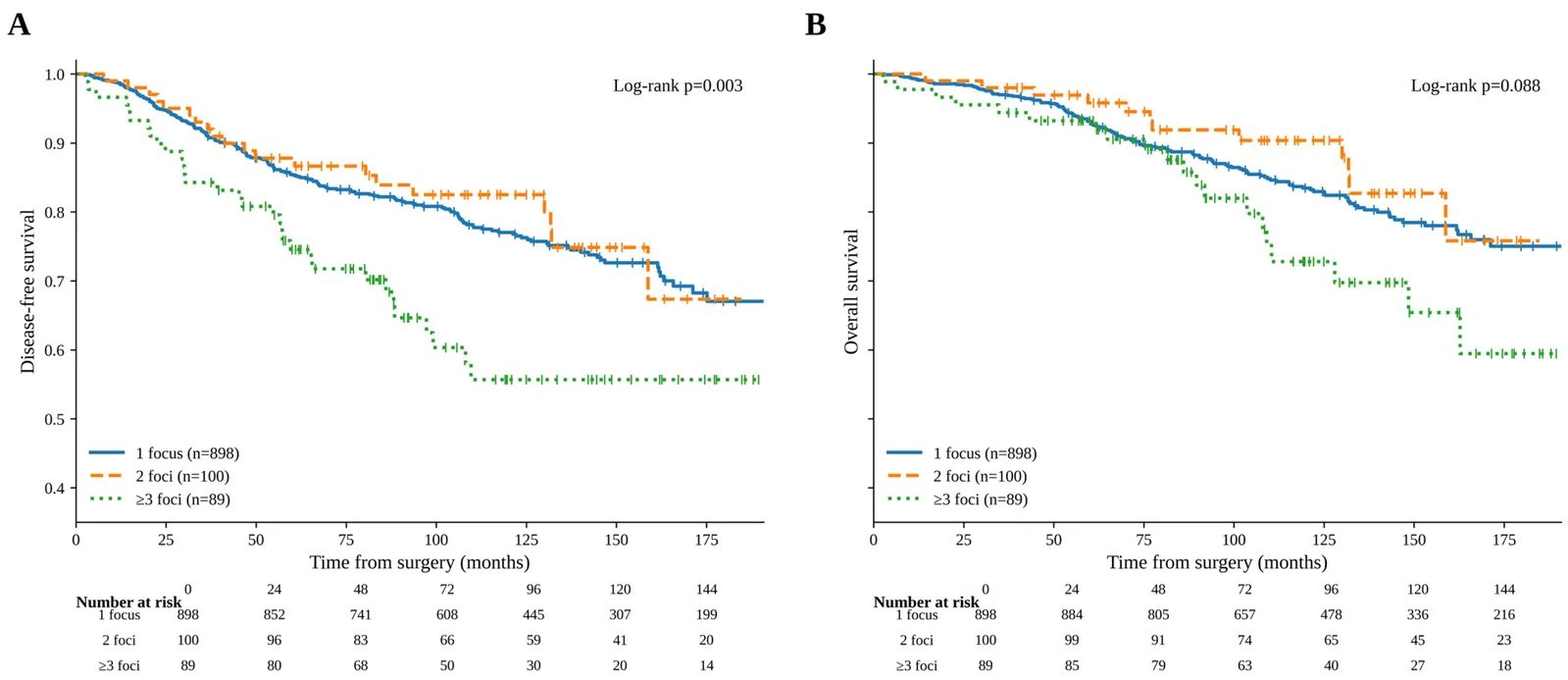
Kaplan–Meier survival curves according to the number of invasive tumor foci. (**A**) Disease-free survival according to the number of invasive tumor foci (log-rank *p* = 0.003). (**B**) Overall survival according to the number of invasive tumor foci (log-rank *p* = 0.088). Censored observations are indicated by tick marks.

The median follow-up was 113.1 months. During follow-up, 250 DFS events and 171 deaths were recorded. DFS events occurred in 198/898 patients (22.0%) with one focus, 20/100 (20.0%) with two foci, and 32/89 (36.0%) with three or more foci. The first DFS events were locoregional recurrence in 16, 2, and 2 patients; distant metastasis in 127, 13, and 28; and death without prior recurrence or metastasis in 55, 5, and 2, respectively. DFS differed according to the number of invasive tumor foci (log-rank *p* = 0.003). The 5-year DFS rates were 85.4% (95% CI 82.8–87.5), 87.8% (95% CI 79.5–92.9), and 74.6% (95% CI 63.9–82.5) in patients with one, two, and three or more foci, respectively. The corresponding 10-year DFS rates were 77.0% (95% CI 73.7–80.0), 82.5% (95% CI 72.9–89.0), and 55.7% (95% CI 42.4–67.1) (Figure 1A).

A statistically significant difference in OS among the three groups was not demonstrated (log-rank *p* = 0.088). The 5-year OS rates were 93.1% (95% CI 91.2–94.6), 95.8% (95% CI 89.2–98.4), and 93.2% (95% CI 85.5–96.9), and the corresponding 10-year rates were 83.4% (95% CI 80.4–86.1), 90.4% (95% CI 81.5–95.1), and 72.8% (95% CI 59.0–82.6), respectively (Figure 1B). In univariable Cox regression, compared with the one-focus group, the HR for OS was 0.74 (95% CI 0.41–1.33; *p* = 0.314) for patients with two foci and 1.55 (95% CI 0.97–2.47; *p* = 0.068) for those with three or more foci.

In univariable Cox regression analysis, three or more invasive tumor foci were associated with poorer DFS (HR 1.87, 95% CI 1.28–2.71; *p* = 0.001), whereas two foci were not (HR 0.89, 95% CI 0.56–1.40; *p* = 0.603). Age, tumor size, histological grade, lymphatic invasion, vascular invasion, and advanced nodal disease were also associated with DFS. In multivariable analysis, three or more invasive tumor foci remained associated with poorer DFS after multivariable adjustment (HR 1.58, 95% CI 1.07–2.33; *p* = 0.022), whereas two foci were not (HR 0.92, 95% CI 0.58–1.47; *p* = 0.735). Advanced nodal disease was also independently associated with poorer DFS (HR 2.47, 95% CI 1.85–3.30; *p* < 0.001). Age, tumor size, grade 3 disease, and HER2 status were independently associated with DFS (Table 5).

After additional adjustment for adjuvant chemotherapy, radiotherapy, endocrine therapy, trastuzumab use, and surgery type, three or more invasive foci remained associated with poorer DFS (HR 1.52, 95% CI 1.03–2.24; *p* = 0.037), whereas two foci were not significantly associated with DFS (HR 0.91, 95% CI 0.57–1.46; *p* = 0.689). The association between HER2 positivity and DFS was no longer statistically significant (HR 0.67, 95% CI 0.36–1.23; *p* = 0.194). In the reduced model including focus number, age, tumor size, histological grade, and N2/N3 disease, three or more foci remained associated with poorer DFS (HR 1.57, 95% CI 1.07–2.30; *p* = 0.020), whereas two foci were not significantly associated with DFS (HR 0.92, 95% CI 0.58–1.46; *p* = 0.724).

In exploratory analyses within each focality group, three or more foci were associated with poorer DFS than two foci in MF disease (unadjusted HR 2.07, 95% CI 1.12–3.82; *p* = 0.020). The estimate was similar in direction in MC disease but was imprecise and not statistically significant (unadjusted HR 2.22, 95% CI 0.55–8.92; *p* = 0.263).

The PR × ln(time) interaction was significant (*p* = 0.002). After including this term, three or more foci remained associated with poorer DFS (HR 1.58, 95% CI 1.07–2.33; *p* = 0.021), whereas two foci were not significantly associated with DFS (HR 0.93, 95% CI 0.59–1.49; *p* = 0.776).

When focus number was analysed as a continuous variable, each additional focus was associated with higher odds of N2/N3 disease (adjusted OR 1.31, 95% CI 1.14–1.50; *p* < 0.001) and poorer DFS (adjusted HR 1.10, 95% CI 1.01–1.20; *p* = 0.038). Restricted cubic spline analyses showed no significant evidence of nonlinearity for either outcome (*p* = 0.574 for N2/N3 and *p* = 0.936 for DFS).

## 4. Discussion

The main finding of this study was that the association between tumor multiplicity and axillary nodal burden was largely confined to patients with three or more invasive foci. Although MF/MC tumors had more frequent axillary involvement than unifocal tumors, patients with two foci had a nodal burden similar to those with a single focus. In contrast, N2/N3 disease was present in 38.2% of patients with three or more foci, and this association remained significant after adjustment for tumor size and other clinicopathological factors.

Higher rates of axillary metastasis in MF/MC breast cancer have been reported in previous studies. Cabioglu et al. and Duraker and Caynak found greater nodal involvement in MF/MC tumors, including within comparable tumor size categories [3,4]. Kanumuri et al. also found multicentricity to be an independent predictor of lymph node positivity [2]. More recently, Burcu et al. found significantly higher axillary lymph node positivity in MF/MC disease in a cohort of 666 patients [10]. In our cohort, however, the increase in nodal burden was mainly seen in patients with three or more invasive foci.

Few studies have considered tumor multiplicity beyond the conventional MF/MC classification. Weissenbacher et al. reported poorer outcomes in MF/MC disease after matching for tumor size, grade, and hormone receptor status and suggested that the number of foci should be considered in risk assessment [6]. Although the number of tumor foci was recorded in some studies, focus number was not evaluated as a graded predictor [11,12]. In our cohort, N2/N3 disease rates were almost identical in patients with one and two foci (18.4% and 19.0%) but increased to 38.2% in those with three or more foci. Two foci were not associated with advanced nodal disease in multivariable analysis, whereas three or more foci were associated with nearly threefold higher odds. Thus, grouping all tumors with two or more foci together may overlook clinically relevant differences within MF/MC disease. The combined MF/MC analysis addressed focus number rather than anatomical distribution and should not be interpreted as evidence that MF and MC are equivalent. Separate exploratory analyses showed associations in the same direction, but the small MC subgroup limited interpretation.

The optimal representation of tumor burden in MF/MC disease remains debated. Coombs and Boyages found that using aggregate tumor diameter reclassified a significant number of multifocal tumors and eliminated the excess nodal positivity observed when tumors were staged according to the largest focus alone [13]. Andea et al. later found that MF/MC tumors still had higher odds of nodal involvement than unifocal tumors of comparable aggregate volume or surface area [14]. Boyages et al. found that aggregate tumor size better accounted for the poorer 10-year breast cancer-specific survival observed in multifocal tumors larger than 20 mm, questioning whether the dominant focus alone always captures tumor burden [15]. Fushimi et al. subsequently reported worse DFS among patients whose pathological T category increased when the diameters of individual invasive foci were summed [16]. More recently, Tong et al. reported that aggregate dimensions upstaged 63 patients with MF/MC disease and that multiplicity was no longer independently associated with lymph node positivity when aggregate rather than largest tumor dimension was used [12]. In contrast, Dal et al., who compared maximum and aggregate diameter, area, and volume, found that T stage based on the largest lesion remained independently associated with OS [17]. These studies do not clearly support replacing the conventional largest-focus approach.

The prognostic impact of MF/MC disease has been less consistent than its association with nodal involvement. A meta-analysis of 31 studies including 15,703 patients found no significant association between MF/MC disease and OS, DFS, breast cancer-specific survival, recurrence-free survival, or local recurrence-free survival when time-to-event estimates were pooled [9]. In contrast, Ustaalioglu et al. reported significantly shorter DFS in patients with MF/MC tumors and found MF/MC disease to be independently associated with DFS, whereas the difference in OS was not statistically significant [18]. In our cohort, DFS differed according to the number of invasive foci. Ten-year DFS was 55.7% in patients with three or more foci compared with 77.0% and 82.5% in those with one and two foci, respectively. Three or more foci were associated with poorer DFS in univariable analysis and remained associated with poorer DFS after adjustment for nodal status and other clinicopathological factors, whereas two foci were not associated with DFS in either analysis. A statistically significant difference in OS among the three groups was not demonstrated. However, the univariable estimate for the ≥3-foci group was in the direction of poorer OS (HR 1.55, 95% CI 0.97–2.47; *p* = 0.068), and the study may have had limited power for this endpoint. DFS estimates were numerically higher in patients with two foci than in those with one focus, although two foci were not significantly associated with DFS in the Cox analyses. The categorical comparisons did not show a clear stepwise worsening of prognosis. Exploratory continuous analyses supported an overall association between focus number and both outcomes, without significant evidence of nonlinearity; these findings do not establish three foci as a distinct prognostic threshold. The distinction between two and three or more foci may partly account for the inconsistent survival results reported in previous studies.

Contemporary data also support breast conservation in appropriately selected patients with multiple ipsilateral tumors. In the prospective ACOSOG Z11102 trial, women with two or three ipsilateral foci treated with breast-conserving surgery and radiotherapy had a 5-year local recurrence rate of 3.1% [19]. A recent systematic review and meta-analysis reported favorable estimated DFS and OS after breast-conserving therapy in MF/MC disease. Although local recurrence was higher than in unifocal tumors overall, this difference was not apparent in more recent studies [20].

Histological and immunohistochemical heterogeneity among individual foci is another feature of MF/MC disease. Dal et al. identified histological or immunohistochemical heterogeneity in 29.2% of their patients, although heterogeneity was not associated with recurrence or survival [21]. Tong et al. also reported discordance among individual foci in pathological type (9.9%), histological grade (4.1%), and molecular subtype (8.3%) [12]. More recently, Wu et al. demonstrated HER2 discordance among individual invasive foci. Discordance was uncommon using the conventional HER2-negative/HER2-positive classification (4.0%) but increased to 23.7% when HER2-null, HER2-ultralow, HER2-low, and HER2-positive categories were considered [22]. The dominant lesion may therefore not always reflect the pathological or biomarker profile of the other invasive foci. Whether this heterogeneity is related to the poorer DFS observed in patients with a greater number of foci remains unclear.

The retrospective, single-center design is a limitation of this study. The group with three or more foci included only 89 patients and 32 DFS events, and several covariates were included in the primary Cox model; the precision of the adjusted estimate is therefore limited. The similar HR observed in the reduced model supports the consistency of the finding but does not eliminate this limitation. Only 29 patients had MC disease, and the DFS estimate in this subgroup should be regarded as exploratory. The small MC group also precluded reliable multivariable analysis within this subgroup. The long inclusion period also encompassed changes in imaging, pathological assessment, and systemic treatment. Although the general pathological sampling approach remained similar throughout the study period, changes in imaging and reporting practices over the long inclusion period may have influenced the detection of additional invasive foci. Clinical nodal status and the type of axillary surgery were not available in the study dataset, preventing analyses by axillary procedure. The association between three or more foci and N2/N3 disease persisted in patients with at least 10 examined lymph nodes, although this restriction does not fully account for differences in axillary staging. Individual measurements of all invasive foci were not consistently available, preventing comparison of focus number with aggregate tumor diameter or volume. Adjustment for the size of the largest focus does not account for total invasive tumor burden, which may partly explain the observed associations with N2/N3 disease and DFS. Ki-67 was unavailable in 27.5% of patients, and the analyses were therefore not adjusted for this marker. Patients receiving neoadjuvant treatment were excluded, which may have introduced selection bias because patients with more extensive MF/MC disease may have been more likely to receive neoadjuvant therapy. The association between three or more foci and poorer DFS persisted after additional adjustment for adjuvant treatments and surgery type. HER2 positivity was no longer significantly associated with DFS in this analysis, and its association in the primary model should therefore be interpreted cautiously. The large cohort, detailed pathological nodal data, long follow-up, and separate evaluation of the number of invasive foci are strengths of the study.

## 5. Conclusions

The greater axillary nodal burden associated with MF/MC breast cancer was mainly observed in patients with three or more invasive foci. Three or more foci were associated with both N2/N3 disease and poorer DFS after adjustment for the measured clinicopathological factors, whereas two foci were not. These findings are hypothesis-generating, as the contribution of total invasive tumor burden could not be assessed. These findings apply to patients who underwent upfront surgery and should not be extrapolated to those receiving neoadjuvant therapy, particularly patients with node-positive, HER2-positive, triple-negative, or extensive disease. Validation in contemporary multicenter cohorts, including patients receiving neoadjuvant therapy, is warranted.

## Figures and Tables

**Table 1:** Clinicopathological characteristics according to tumor focality.

Parameters	Unifocal (*n* = 898), *n* (%)	MF/MC (*n* = 189), *n* (%)	*p*-Value
Age, years, median	51 (44–61)	48 (42–58)	0.017
Menopausal status			0.001
Premenopausal	413 (46.0)	111 (58.7)	
Postmenopausal	485 (54.0)	78 (41.3)	
Laterality			0.032 *
Right	463 (51.6)	97 (51.3)	
Left	434 (48.3)	89 (47.1)	
Bilateral	1 (0.1)	3 (1.6)	
Tumor localization			0.150
Upper outer quadrant	539 (60.0)	105 (55.6)	
Lower outer quadrant	100 (11.1)	23 (12.2)	
Lower inner quadrant	78 (8.7)	27 (14.3)	
Upper inner quadrant	120 (13.4)	25 (13.2)	
Retroareolar	61 (6.8)	9 (4.8)	
Surgical procedure			0.003
Breast-conserving surgery	341 (38.0)	50 (26.5)	
Modified radical mastectomy	557 (62.0)	139 (73.5)	
Histological type			0.003
Invasive ductal carcinoma	714 (79.5)	136 (72.0)	
Invasive lobular carcinoma	53 (5.9)	24 (12.7)	
Other	131 (14.6)	29 (15.3)	
Tumor size, cm, median	2.4 (1.5–3.3)	2.3 (1.5–3.2)	0.201
Histological grade			0.463
Grade 1	193 (21.5)	36 (19.0)	
Grade 2	467 (52.0)	95 (50.3)	
Grade 3	238 (26.5)	58 (30.7)	
Lymphatic invasion			0.964
Negative	529 (58.9)	111 (58.7)	
Positive	369 (41.1)	78 (41.3)	
Vascular invasion			0.264
Negative	557 (62.0)	109 (57.7)	
Positive	341 (38.0)	80 (42.3)	
Perineural invasion			0.190
Negative	700 (78.0)	139 (73.5)	
Positive	198 (22.0)	50 (26.5)	
DCIS component			0.001
Absent	330 (36.7)	46 (24.3)	
Present	568 (63.3)	143 (75.7)	
LCIS component			0.053
Absent	839 (93.4)	169 (89.4)	
Present	59 (6.6)	20 (10.6)	
ER status			0.500
Negative	131 (14.6)	24 (12.7)	
Positive	767 (85.4)	165 (87.3)	
PR status			0.800
Negative	239 (26.6)	52 (27.5)	
Positive	659 (73.4)	137 (72.5)	
HER2 status			0.022
Negative	601 (66.9)	110 (58.2)	
Positive	297 (33.1)	79 (41.8)	
Ki-67, %, median	25 (13–40)	20.5 (10–40)	0.200

Data are presented as *n* (%) unless otherwise indicated. Continuous variables are presented as median (25th–75th percentile). * Fisher–Freeman–Halton exact test. MF, multifocal; MC, multicentric; DCIS, ductal carcinoma in situ; LCIS, lobular carcinoma in situ; ER, estrogen receptor; PR, progesterone receptor; HER2, human epidermal growth factor receptor 2.

**Table 2:** Axillary nodal burden according to tumor focality.

Parameters	Unifocal (*n* = 898), *n* (%)	MF/MC (*n* = 189), *n* (%)	*p*-Value
Number of examined lymph nodes, median	17 (11–24)	17 (12–24)	0.696
Number of metastatic lymph nodes, median	1 (0–2)	1 (0–4)	0.002
Axillary lymph node involvement			0.020
Negative	440 (49.0)	75 (39.7)	
Positive	458 (51.0)	114 (60.3)	
Pathological nodal stage			0.016
N0	440 (49.0)	75 (39.7)	
N1	293 (32.6)	61 (32.3)	
N2	92 (10.2)	28 (14.8)	
N3	73 (8.1)	25 (13.2)	
Advanced nodal disease			0.003
N0/N1	733 (81.6)	136 (72.0)	
N2/N3	165 (18.4)	53 (28.0)	
Extracapsular extension			0.012
Absent	674 (75.1)	125 (66.1)	
Present	224 (24.9)	64 (33.9)	

Data are presented as *n* (%) unless otherwise indicated. Continuous variables are presented as median (25th–75th percentile). MF, multifocal; MC, multicentric.

**Table 3:** Axillary nodal burden according to the number of invasive tumor foci.

Parameters	1 Focus (*n* = 898), *n* (%)	2 Foci (*n* = 100), *n* (%)	≥3 Foci (*n* = 89), *n* (%)	*p*-Value
Number of examined lymph nodes, median	17 (11–24)	15.5 (11–23.3)	18 (13–24)	0.266
Number of metastatic lymph nodes, median	1 (0–2)	1 (0–3)	2 (0–8)	<0.001
Axillary lymph node involvement				0.012
Negative	440 (49.0)	46 (46.0)	29 (32.6)	
Positive	458 (51.0)	54 (54.0)	60 (67.4)	
Pathological nodal stage				<0.001
N0	440 (49.0)	46 (46.0)	29 (32.6)	
N1	293 (32.6)	35 (35.0)	26 (29.2)	
N2	92 (10.2)	13 (13.0)	15 (16.9)	
N3	73 (8.1)	6 (6.0)	19 (21.3)	
Advanced nodal disease				<0.001
N0/N1	733 (81.6)	81 (81.0)	55 (61.8)	
N2/N3	165 (18.4)	19 (19.0)	34 (38.2)	
Extracapsular extension				<0.001
Absent	674 (75.1)	76 (76.0)	49 (55.1)	
Present	224 (24.9)	24 (24.0)	40 (44.9)	

Data are presented as *n* (%) unless otherwise indicated. Continuous variables are presented as median (25th–75th percentile).

**Table 4:** Univariable and multivariable logistic regression analysis for advanced axillary nodal burden.

Variable	Univariable OR (95% CI)	*p*-Value	Multivariable OR (95% CI)	*p*-Value
Number of invasive tumor foci				
1 focus	Reference		Reference	
2 foci	1.04 (0.61–1.77)	0.878	1.29 (0.73–2.29)	0.383
≥3 foci	2.75 (1.73–4.35)	<0.001	2.81 (1.65–4.79)	<0.001
Age, per year	1.00 (0.98–1.01)	0.571	1.00 (0.99–1.02)	0.791
Tumor size, per cm	1.34 (1.23–1.47)	<0.001	1.22 (1.11–1.34)	<0.001
Histological grade				
Grade 1	Reference		Reference	
Grade 2	3.79 (2.23–6.45)	<0.001	2.55 (1.43–4.54)	0.001
Grade 3	3.86 (2.20–6.78)	<0.001	2.05 (1.10–3.81)	0.024
Histological type				
Ductal	Reference		Reference	
Lobular	1.02 (0.57–1.81)	0.949	0.94 (0.49–1.83)	0.863
Other	0.82 (0.53–1.28)	0.389	0.80 (0.49–1.30)	0.372
Lymphatic invasion, positive	6.48 (4.61–9.11)	<0.001	3.31 (1.69–6.48)	<0.001
Vascular invasion, positive	5.90 (4.24–8.20)	<0.001	1.80 (0.94–3.46)	0.077
ER status, positive	0.92 (0.60–1.39)	0.678	1.76 (1.00–3.10)	0.051
PR status, positive	0.68 (0.50–0.94)	0.020	0.70 (0.45–1.09)	0.110
HER2 status, positive	1.50 (1.11–2.04)	0.009	1.20 (0.85–1.70)	0.294

Advanced axillary nodal burden was defined as N2/N3 disease. OR, odds ratio; CI, confidence interval; ER, estrogen receptor; PR, progesterone receptor; HER2, human epidermal growth factor receptor 2.

**Table 5:** Univariable and multivariable Cox proportional hazards regression analysis for disease-free survival.

Variable	Univariable HR (95% CI)	*p*-Value	Multivariable HR (95% CI)	*p*-Value
Number of invasive tumor foci				
1 focus	Reference		Reference	
2 foci	0.89 (0.56–1.40)	0.603	0.92 (0.58–1.47)	0.735
≥3 foci	1.87 (1.28–2.71)	0.001	1.58 (1.07–2.33)	0.022
Age, per year	1.02 (1.00–1.03)	0.007	1.02 (1.01–1.03)	0.004
Tumor size, per cm	1.18 (1.11–1.25)	<0.001	1.10 (1.03–1.17)	0.007
Histological grade				
Grade 1	Reference		Reference	
Grade 2	1.72 (1.20–2.48)	0.003	1.31 (0.90–1.91)	0.157
Grade 3	1.92 (1.30–2.85)	0.001	1.52 (1.01–2.30)	0.047
Histological type				
Ductal	Reference		Reference	
Lobular	1.17 (0.73–1.87)	0.520	0.98 (0.61–1.59)	0.935
Other	1.13 (0.79–1.60)	0.501	1.08 (0.76–1.54)	0.658
Lymphatic invasion, positive	2.12 (1.65–2.72)	<0.001	1.09 (0.61–1.95)	0.759
Vascular invasion, positive	2.18 (1.70–2.80)	<0.001	1.34 (0.76–2.38)	0.316
ER status, positive	0.93 (0.66–1.31)	0.677	1.02 (0.65–1.60)	0.936
PR status, positive	0.87 (0.66–1.14)	0.299	0.95 (0.67–1.35)	0.767
HER2 status, positive	0.83 (0.63–1.09)	0.171	0.67 (0.51–0.89)	0.006
Advanced nodal disease (N2/N3)	3.26 (2.53–4.20)	<0.001	2.47 (1.85–3.30)	<0.001

Disease-free survival was defined as the time from definitive breast surgery to the first occurrence of locoregional recurrence, distant metastasis, or death from any cause. The proportional hazards assumption was violated for PR status. A sensitivity analysis incorporating a time-dependent PR term did not materially alter the association between tumor focality and DFS. HR, hazard ratio; CI, confidence interval; ER, estrogen receptor; PR, progesterone receptor; HER2, human epidermal growth factor receptor 2.

## Data Availability

The data presented in this study are available from the corresponding author upon reasonable request.

## Abbreviations

CI, confidence interval; DCIS, ductal carcinoma in situ; DFS, disease-free survival; ER, estrogen receptor; FISH, fluorescence in situ hybridization; HER2, human epidermal growth factor receptor 2; HR, hazard ratio; IHC, immunohistochemistry; IQR, interquartile range; LCIS, lobular carcinoma in situ; MC, multicentric; MF, multifocal; MF/MC, multifocal/multicentric; OR, odds ratio; OS, overall survival; PR, progesterone receptor.
